# Action Semantic Deficits and Impaired Motor Skills in Autistic Adults Without Intellectual Impairment

**DOI:** 10.3389/fnhum.2019.00256

**Published:** 2019-07-25

**Authors:** Josephina Hillus, Rachel Moseley, Stefan Roepke, Bettina Mohr

**Affiliations:** ^1^Department of Psychiatry and Psychotherapy, Charité Universitätsmedizin, Campus Benjamin Franklin, Berlin, Germany; ^2^Department of Psychology, Bournemouth University, Poole, United Kingdom

**Keywords:** autism, semantic processing, language, motor, action words

## Abstract

Several studies indicate the functional importance of the motor cortex for higher cognition, language and semantic processing, and place the neural substrate of these processes in sensorimotor action-perception circuits linking motor, sensory and perisylvian language regions. Interestingly, in individuals with autism spectrum disorder (ASD), semantic processing of action and emotion words seems to be impaired and is associated with hypoactivity of the motor cortex during semantic processing. In this study, the relationship between semantic processing, fine motor skills and clinical symptoms was investigated in 19 individuals with ASD and 22 typically-developing matched controls. Participants completed two semantic decision tasks involving words from different semantic categories, a test of alexithymia (the Toronto Alexithymia Scale), and a test of fine motor skills (the Purdue Pegboard Test). A significant Group × Word Category interaction in accuracy (*p* < 0.05) demonstrated impaired semantic processing for action words, but not object words in the autistic group. There was no significant group difference when processing abstract emotional words or abstract neutral words. Moreover, our study revealed deficits in fine motor skills as well as evidence for alexithymia in the ASD group, but not in neurotypical controls. However, these motor deficits did not correlate significantly with impairments in action-semantic processing. We interpret the data in terms of an underlying dysfunction of the action-perception system in ASD and its specific impact on semantic language processing.

## Introduction

Neuroscientific research on “embodied cognition” postulates that higher cognitive processes, such as language, thought and reasoning, are functionally (and possibly structurally) interwoven with lower-level sensory and motor functions (Gallese and Lakoff, 2005; Barsalou, 2010). To this end, recent empirical evidence from behavioral and neuroimaging studies demonstrate that the motor cortex serves an important function for language processing, particularly during semantic processing (Pulvermüller, 1999; Pulvermüller et al., 2005; Moseley et al., 2013). More specifically, semantic processing of words associated with actions and motor movements activate the motor cortex somatotopically (Hauk et al., 2004; Pulvermüller and Fadiga, 2010; Moseley et al., 2012), which may be explained on the basis of the formation and activation of sensorimotor action-perception circuits comprising neurons in the motor cortex, in sensory cortices and in perisylvian language areas (Pulvermüller and Fadiga, 2010; Pulvermüller, 2012; Pulvermüller et al., 2014). Interestingly, recent data reveal a specific weakness in the processing of action-related words in clinical populations who have motor impairments (Boulenger et al., 2009; Bak and Chandran, 2012; Fernandino et al., 2013a,b; Cardona et al., 2014; Kemmerer, 2014; Desai et al., 2015). Specific impairments in action-semantic processing have also been reported in individuals with autism spectrum disorder (ASD), a neurodevelopmental syndrome characterized by problems with social interaction, communication and language, and, importantly, by dysfunction in motor behavior [American Psychiatric Association (APA), (2000)]. The motor deficits seen in ASD, ranging from differences in gait, fine motor skills, posture and coordination, are pervasive across the spectrum, occur in individuals with and without intellectual impairment, and are among the earliest symptoms to appear (Leary and Hill, 1996; Jansiewicz et al., 2006; Dziuk et al., 2007; Ming et al., 2007; Moseley and Pulvermüller, 2018). Unsurprisingly, abnormalities in structural and functional connectivity have been reported within and between primary motor cortex and other cortical regions in ASD (Mostofsky et al., 2007, 2009; McCleery et al., 2013; Floris et al., 2016; Thompson et al., 2017), as have differences in gray matter volume (Duffield et al., 2013; Mahajan et al., 2016), thus suggesting that the action-semantic deficit in this group is comparable to that seen in other populations with disease or damage to the motor system.

In the past, cognitive theories of ASD have centered around the archetypal “autistic triad” of deficits in social interaction, social communication and social imagination (Wing and Gould, 1979); as such, obvious motor impairments have been traditionally regarded as secondary and consequently neglected in research. To date, few studies on autism have focused on highlighting the functional relationship between motor symptoms and difficulties in higher-order cognitive functions, which include action-related cognition (e.g., imitation and gesturing). The functional link between an observed action and its corresponding motor program may be required to perform a self-generated movement and has been attributed to the *mirror neuron system* (MNS) which is posited to exist across primary and premotor cortex, somatosensory cortex, and parietal cortex. Responsive to both action perception and action execution, mirror neurons appear to be a quintessential type of multimodal “information-mixing” neuron, and a crucial element in binding motor areas to sensory and perisylvian language areas in action-perception circuits (Moseley and Pulvermüller, 2018). A number of studies consequently suggest that the MNS may be relevant in action perception, imitation, prediction of goals and intentions, as well as in social cognition and language (Iacoboni, 2009; Rizzolatti and Sinigaglia, 2010).

Previous studies have demonstrated functional impairments and neuronal hypoactivity of the MNS in autism (Nishitani et al., 2004; Oberman et al., 2005; Iacoboni and Dapretto, 2006; Bernier et al., 2007; Cattaneo et al., 2007; Honaga et al., 2010; Rizzolatti and Fabbri-Destro, 2010; McCleery et al., 2013; Wadsworth et al., 2017). These are consequently posited as the neuronal substrate of behavioral deficits in action-related cognition, which are interpreted as a consequence of dysfunctional action-perception mapping. This is manifest in impaired semantic processing for action but not object words in autistic individuals without intellectual disability, an impairment which correlated with reduced activation in cortical motor regions during action-word processing (Moseley et al., 2014, 2015; Moseley and Pulvermüller, 2018). Moreover, further studies in this clinical group revealed hypoactivation in motor as well as in limbic areas during processing of abstract emotional words (Moseley et al., 2012, 2015), which other studies have shown to be a notable challenge for autistic people. These findings have been interpreted on the basis that both of these semantic categories (action and emotion words) typically involve the activation of premotor and motor action-perception networks during learning and require this activity for efficient, optimal comprehension. This is consistent with the recent suggestion that hypoactivity of the motor cortex could also be one of the reasons for deficits in the socio-communicative and emotional-affective domain in ASD (Mody et al., 2017). Functional impairments between the motor cortex and perisylvian language regions may thus be related to social-communicative and emotional-affective deficits in individuals with ASD, as the development of semantic concepts would be mandatory for verbally expressing and understanding emotions in oneself and others.

A different theoretical approach explains reduced comprehension of emotional stimuli in ASD in terms of alexithymia, a difficulty in expressing and identifying one’s own emotional states or feelings (Silani et al., 2008; Milosavljevic et al., 2016; Gaigg et al., 2018). However, a point of convergence might be that alexithymia itself may be (partially) caused by dysfunctional semantic processing of emotion words, which might, in turn, be linked to impaired action-perception circuits involving motor and limbic regions. Emotions clearly influence the style in which an action is performed, and thus predictably, the same multimodal mirror neurons of frontal-motor and parietal cortex are sensitive to different emotional states underpinning the same observed action (Di Cesare et al., 2015). This suggests the importance of the motor system in perceiving emotional states.

Previous studies demonstrated atypical brain activity in motor systems whilst autistic people read action and emotion words (Moseley et al., 2014, 2015), which also seems to be linked to a behavioral slowness in processing action words (Moseley et al., 2013). The next piece of this puzzle, however, remains missing: the link between language impairment for action and emotion words and *movement* impairment. To clarify this functional link, our study aimed to investigate the relationship between semantic processing of action and emotion words, fine and gross motor skills, and clinical symptoms in individuals with ASD and in typically-developed (TD) controls. In line with previous research with autistic participants, we predicted a specific processing deficit for action and emotion words but no groups differences for other word categories. We hypothesized that deficits in motor skills in individuals with ASD would be associated with clinical symptoms and impairments in processing these specific word categories.

## Materials and Methods

### Participants

Nineteen autistic adults without intellectual disability (seven women) and 23 TD controls (nine women) were recruited for the study. One control participant had to be excluded from the final analysis due to poor task performance in the semantic decision task; therefore, the final data set comprised 19 ASD and 22 TD participants. All participants had normal or corrected-to-normal vision. In the control group, none of the participants had a history of psychiatric illness. Three participants in the ASD group took antidepressants.

The groups were matched for age, education, non-verbal IQ (measured by the LPS-3, Horn, 1983), and handedness (measured by the Edinburgh Handedness Inventory, Oldfield, 1971). Except for two participants in the ASD group, all participants were right-handed with a matched laterality-quotient (LQ). All participants were monolingual, native speakers of German. More information on both groups can be found in Table 1.

**Table 1:** Means and standard deviations (SD, in brackets) of demographic and clinical variables used to match the autism spectrum disorder (ASD) and TD groups.

	ASD group *N* = 19	TD control group *N* = 22	Statistical group difference
Age (years)	39.00 (11.20)	36.59 (7.55)	n.s. (*p* = 0.4)
Education (years)	12.00 (1.52)	12.73 (0.88)	n.s. (*p* = 0.06)
IQ (LPS-3)	117.76 (9.75)	112.96 (8.72)	n.s. (*p* = 0.1)
Laterality Quotient (LQ)	79.79 (16.09)	88.18 (15.31)	n.s. (*p* = 0.09)
Autism-Spectrum Quotient (AQ)	39.05 (6.62)	11.59 (4.02)	*p* < 0.001

*Between-group differences were calculated by independent t-tests (p-values are in brackets; n.s. indicates non-significant result). Groups did not differ on any variable except on the AQ*.

All ASD participants were diagnosed and recruited from the Autism Outpatient Clinic at the Charité University Medical School, Benjamin Franklin Campus, Berlin, Germany. Autism-specific diagnostic instruments were used for diagnosis, including the Autism Diagnostic Observation Schedule (ADOS; Lord et al., 2002) and a semi-structured clinical interview based on ASD criteria in the Diagnostic and Statistical Manual of Mental Disorders, 4th edition [DSM-IV; American Psychiatric Association (APA), (2000)]. If a parent was available—which was the case in 66% of all ASD patients—the Autism Diagnostic Interview-Revised (Lord et al., 1994) was conducted. Final diagnoses were established by expert consensus taking into account clinical interviews and scale assessments. A patient was diagnosed with ASD when scores on both the ADOS and the ADI-R exceeded the cut-off for autism spectrum or autism and all required DSM-IV criteria of the clinical interview were fulfilled. For the 33% of patients whose parents were not available for the ADI-R interview, an ASD diagnosis was given when all required criteria of the ADOS and the clinical interview were met and the patient provided sufficient examples that the autistic symptoms already existed in childhood.

The mean score of the ASD group on the Autism-Spectrum Quotient (AQ: Baron-Cohen et al., 2001) was 39.1 (SD: 6.6) compared to a mean score of 11.59 (SD: 4.020) in the control group: as expected, a significantly higher average score (*t*_(39)_ = 16.302, *p* < 0.001). All but one participant in the ASD group scored above 26, which is considered as the general cut-off point for diagnosable autism (Woodbury-Smith et al., 2005).

### Neuropsychological and Clinical Assessment

#### Leistungsprüfsystem-Test, Subtest 3

The *Leistungsprüfsystem-Test, Subtest 3* (Horn, 1983) was carried out with all participants to assess non-verbal IQ. Handedness was measured by the *Laterality Quotient*, assessed by the *Edinburgh Handedness Inventory* (Oldfield, 1971).

#### Purdue Pegboard Test

The Purdue Pegboard Test was used in both groups to assess manual dexterity, manual coordination and fingertip skills (Tiffin and Asher, 1948). The test consists of a board with two parallel rows of 25 holes running vertically. Participants were asked to use their right hand to put as many of the cylindrical metal pegs as possible in the right-sided row within 30 s; the same procedure was then followed for the left hand with the left-sided row. In a third condition which combined the two previous trials, participants had to simultaneously place the pegs within the right- and left-sided rows with their right and left hands respectively. In a fourth condition, as many “assemblies” as possible, consisting of different objects, had to be built within 60 s.

#### Trailmaking Test (Parts A and B)

The Trailmaking Test (TMT; Parts A and B) is a neuropsychological test to measure attention, processing speed and executive functions (Tombaugh, 2004). This test was performed with the ASD group only in order to assess psychomotor speed and attention (Part A) as well as executive function (Part B).

### Clinical Questionnaires

All participants filled out the Autism-Spectrum Quotient (AQ) and the Toronto Alexithymia Scale 26 (TAS-26; Taylor et al., 1985). The AQ measures the degree of autistic traits whereby higher scores indicate a higher degree of autistic traits (Baron-Cohen et al., 2001). This most popular dimensional measure of autistic traits has been extensively used and validated both in the general population and those with diagnosed autism (Hurst et al., 2007; Hoekstra et al., 2008; Ruzich et al., 2015, 2016; Stevenson and Hart, 2017), where it boasts sound psychometric properties.

Alexithymia is popularly understood as a dimensional construct (Keefer et al., 2019) which is most commonly measured with the TAS-26. This scale comprises three subscales assessing the difficulties describing emotions (scale 1), difficulties identifying one’s own emotions (scale 2), and the tendency to think in an externally-oriented way (scale 3).

Furthermore, all ASD participants filled out the Empathy Quotient (EQ; Baron-Cohen et al., 2014) and the Systemizing Quotient-R (SQ-R; Baron-Cohen et al., 2003; Wheelwright et al., 2006). The EQ measures the capacity for empathy, whereby a lower score indicates reduced empathy. The SQ-R measures the capacity for recognizing patterns and the tendency to “systemize,” to see the world in terms of logical rules and systems and to try to impose these in life, whereby higher scores reflect greater tendency to systemizing. Developed by the same group as the AQ, EQ scores tend to be lower and SQ-R scores higher in autistic individuals, and both short forms of the original tests showed good psychometric properties (Wheelwright et al., 2006).

In an additional, self-designed questionnaire, the MOSES-Test (“Motor Skills in Everyday Situations”), participants had to self-assess their motor skills in everyday situations on a four-point Likert scale employing 12 statements such as “I can easily catch or throw a ball,” or “I have no difficulties riding a bike.” Possible scores ranged from 0 (“I completely agree”) to 3 (“I completely disagree”). If the statements concerned difficulties (“I have difficulties in climbing stairs”), then scores ranged from 3 (“I completely agree”) to 0 (“I completely disagree”). With an upper limit of 36, higher scores on this questionnaire suggest more difficulties in gross motor skills. The MOSES-Test can be found in the Supplementary Materials.

### Semantic Decision Tasks

#### Stimuli

In the first semantic decision task (SDT1; see details below), 90 action-related words {30 face-related [e.g., “BEISSEN” (“TO BITE”)], 30 hand-related [e.g., “MALEN” (“TO PAINT”)], 30 foot-related [e.g., “LAUFEN” (“TO WALK”)]} and 90 object-related words {30 animal words [e.g., “MAUS” (“MOUSE”)], 30 tool words [e.g., “HAMMER” (“HAMMER”)], 30 food words [e.g., “KUCHEN” (“CAKE”)]} were included.

In the second semantic decision task (SDT2; details below), we included 30 abstract emotional words [e.g., “FREUDE” (“JOY”)] and 30 abstract neutral words [e.g., “PLANEN” (“TO PLAN”)]. Abstract emotional words consisted of verbs and nouns associated with emotions, and the abstract neutral word category included verbs and nouns referring to emotionally neutral concepts or cognitions. Words were selected and matched as carefully as possible based on psycholinguistic properties such as word length and word frequency according to the CELEX database (Baayen et al., 1993).

Before conducting this experiment, a semantic rating study was carried out with 10 typically-developing participants who did not take part in the main experiment. This pre-experiment rating study was conducted to differentiate the selected word categories with respect to their semantic properties (see also Hauk et al., 2004; Moseley et al., 2015). Study participants rated all words with regards to semantic features such as concreteness, arousal, valence, emotion-relatedness and action-relatedness. Psycholinguistic variables and semantic ratings for the four major stimulus categories (action-, object-, abstract emotional-, abstract internal words) used in SDT 1 and 2 are displayed in the Supplementary Materials.

#### Procedure

All participants performed two separate and independent semantic decision tasks (SDT1 and SDT2) using E-prime software (Psychology Software Tools, Inc., Sharpsburg, PA, USA, RRID:SCR_009567). The first SDT1 was carried out employing action- and object-related words; the second SDT2 task used abstract emotional and abstract neutral words. Each semantic decision task lasted 10 min, with a break given in between.

Participants were seated approximately 60 cm distance from the computer screen while words appeared on a white background in uppercase, black bold print. All participants were asked to decide as fast and accurately as possible if the presented words were related to human actions or to objects (in SDT1) or, in the second task (SDT2), whether the words were related to emotional or non-emotional abstract concepts. Participants indicated their semantic judgments by pressing one of two keys on a computer keyboard with the index and middle fingers of their right hand. The assignment of keys was counterbalanced between participants. After a fixation cross was shown at central location for 250 ms, words were presented tachistoscopically for 150 ms in a pseudorandomized order. Participants were shown the same words with each word being only shown once to each participant. After the offset of the word, a blank screen was shown until the participant made a decision, or until 2,500 ms had passed without a response, at which point the screen returned to the fixation cross. The stimulus onset asynchrony (SOA) was 2,500 ms. Instead of using their right hand, the two left-handed participants used the index and middle finger of their left hand to perform the SDTs.

### Data Analysis

All data was analyzed using SPSS version 24.0 (RRID:SCR_002865). Independent *t*-tests were used to compare means of demographic variables, neuropsychological tests and clinical questionnaires.

For each participant, we derived mean reaction times and accuracy scores for each word category (action words and object words from SDT1, emotional and non-emotional abstract words from SDT2): this was done by averaging reaction times across all individual words in that category. Each word within a category received either a score of 1 (reflecting correct categorization) or 0 (reflecting that the participant had incorrectly categorized the word or failed to respond). For each participant, the means across these accuracy scores were then transformed into a percentage accuracy for each word category. As such, a mean accuracy score and a mean reaction time score for the action, object, abstract emotional and abstract non-emotional word categories were entered into SPSS for each participant.

To compare reaction times and accuracies of both groups for statistically significant differences, we performed four 2 × 2 mixed design repeated measures analysis of variances (ANOVAs). In each ANOVA, the between factor “Group” (two levels: ASD vs. control) and the within factor “Word Category” [two levels: action words vs. object words (SDT1), emotional vs. non-emotional abstract words (SDT2)] were included.

As concepts, tools and the words denoting them are known to evoke activity in motor regions which are associated with their action affordances, i.e., the actions associated with their use (Chao and Martin, 2000; Carota et al., 2012). As such, these “object-related” tool words tend to be semantically related not only to visual objects but also to specific actions (for instance, a fork to eating). In order to control for this potential “action-relatedness” of the tool word category, we conducted another ANOVA in which tool words were excluded from the analysis. *Post hoc* planned comparisons were conducted with subsequent Bonferroni corrections.

A Pearson correlation was computed for each group separately to assess the relationship between accuracy and latency for each word category in the semantic decision tasks and other variables (AQ, TAS-26, EQ, SQ-R and MOSES-Test). No outlier removal procedure was applied as none of the individual data sets exceeded the mean group values by more than two standard deviations.

## Results

### Neuropsychological and Clinical Assessment

#### Purdue Pegboard

*T*-tests revealed significant differences between the two groups in the first three conditions of the Purdue Pegboard Test (PPB), but not in the fourth “assembly” condition. In comparison to the control group, the ASD group placed significantly fewer pegs with their right hands, left hands and with both hands simultaneously, thus demonstrating impaired fine motor skills (see Table 2).

**Table 2:** Means, standard deviations (in brackets) and statistical group comparisons in the Purdue Pegboard (PPB) Test.

	ASD group *N* = 19	Control group *N* = 22	Statistical testing (*t*)
PPB right	14.16 (1.53)	15.77 (1.51)	*p* < 0.01
PPB left	13.42 (2.38)	14.82 (1.43)	*p* < 0.05
PPB both	11.47 (1.57)	12.41 (1.26)	*p* < 0.05
PPB Assembly	34.74 (7.43)	36.41 (6.68)	n.s. (*p* = 0.45)

*Statistically significant effects are indicated by p-values; n.s. indicates non-significant difference*.

#### Trailmaking Test A and B

We conducted the TMT A and B only for the ASD group and found a mean of 22.05 s (SD: 7.50) in the TMT A and a mean of 49.58 s (SD: 17.58) in the TMT B, indicating unimpaired performance in the range of norms from healthy participants as stated in the test.

### Clinical Questionnaires

#### Toronto-Alexithymia-Scale-26

*T*-tests showed a significant difference between the ASD group and the TD group in overall TAS-26 scores (see Table 3) and in all three sub-scales.

**Table 3:** Means, standard deviations (in brackets) and statistical group comparisons in the TAS-26 questionnaire.

	ASD group *N* = 19	Control group *N* = 22	Statistical testing (*t*)
TAS-26	49.00 (10.29)	38.09 (5.97)	*p* < 0.001
TAS-26 (Scale 1)	18.53 (6.51)	12.09 (2.94)	*p* < 0.001
TAS-26 (Scale 2)	17.79 (4.34)	11.64 (3.65)	*p* < 0.001
TAS-26 (Scale 3)	12.68 (2.81)	14.36 (2.57)	*p* < 0.05

*Statistically significant effects are indicated by p-values*.

#### EQ and SQ-R

The Empathy Questionnaire (EQ) and the Systemizing Questionnaire Revised (SQ-R) were only filled out by the ASD group. The mean score on the SQ-R was 79.21 (SD: 22.837). The mean score of the EQ was 13.89 (SD: 5.597) which is comparable (even slightly lower) than the empathy scores seen in the autistic sample of the original and certainly under the recommended cut-off score of 30, which allowed the authors to distinguish 81% of their autistic sample (Baron-Cohen and Wheelwright, 2004).

#### MOSES-Test

A *t*-test revealed a significant difference in the overall MOSES-score between the ASD group and the control group (*p* < 0.001). The ASD group scored significantly higher with a mean score of 14.53 (SD: 6.851) compared to a mean score of 4.50 (SD: 2.956) in the control group, indicating more motor difficulties in everyday life situations.

### Semantic Decision Tasks

#### SDT1: Action Words vs. Object Words

**Figure 1:**
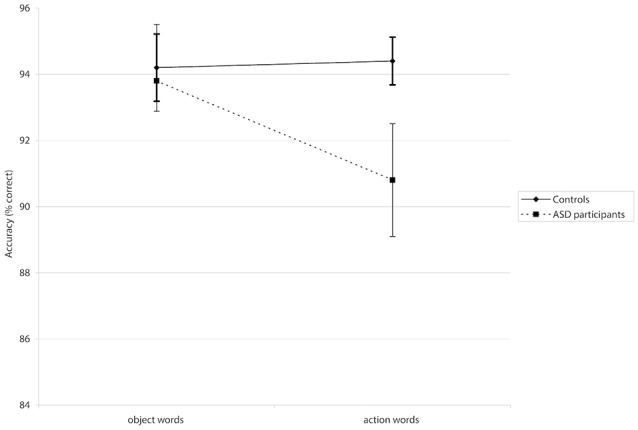
The *Word Category × Group* interaction is displayed for the semantic decision task in which action words and object words were presented. The error bars show the standard error of the mean. Autism spectrum disorder (ASD) participants showed significantly lower accuracy specifically for action words than controls. There was no sign. difference between both groups when processing object words.

A mixed-design repeated measures ANOVA revealed a significant *Group × Word Category* interaction for accuracy (*F*_(1,39)_ = 4.01, *p* < 0.05, $\eta_{\text{p}}^{2}$ = 0.093; see Figure 1). *Post hoc* analyses using pairwise comparisons (Bonferroni-corrected) showed that participants in the ASD group made significantly more errors when presented with action words than they did to object words (*p* < 0.05). This interaction did not show significance in the latency analysis (*F*_(1,39)_ = 0.0001, *p* = 0.985, $\eta_{\text{p}}^{2}$ = 0.0003). There was no significant main effect of *Group* in accuracy *F*_(1,39)_ = 2.42, *p* = 0.128, $\eta_{\text{p}}^{2}$ = 0.06) or latency *F*_(1,39)_ = 0.88, *p* = 0.355, $\eta_{\text{p}}^{2}$ = 0.02), suggesting that where differences did appear, they were associated with particular word categories rather than generally poorer or slower processing. However, a significant main effect of *Word Category* in the latency analysis (*F*_(1,39)_ = 27.15, *p* < 0.001, $\eta_{\text{p}}^{2}$ = 0.41) suggested that *all* participants were slower to process action words; there was a non-significant tendency for them to be less accurate for action words, too (*F*_(1,39)_ = 2.87, *p* = 0.098, $\eta_{\text{p}}^{2}$ = 0.07). Means for accuracies and latencies are presented in Table 4.

**Table 4:** Means and standard deviations (in brackets) for latencies and accuracies.

	ASD group	
**I Action words—Object words**
Reaction time (ms)	630.09 (188)	590.26 (121)
Action words
Reaction time (ms)	573.58 (134)	533.34 (115)
Object words
Accuracy (%)	90.8 (7.4)	94.4 (3.1)
Action words
Accuracy (%)	93.8 (4.0)	94.2 (4.4)
Object words
**II Abstract emotional words—Abstract neutral words**
Reaction time (ms)	816.90 (379)	618.11 (136)
Abstract emotional words
Reaction time (ms)	885.61 (374)	774.62 (208)
Abstract neutral words
Accuracy (%)	91.90 (9.4)	95.80 (4.4)
Abstract emotional words
Accuracy (%)	81.70 (14.5)	90.70 (8.1)
Abstract neutral words		

Furthermore, sub-categories of object and action words were investigated in *post hoc* analyses applying Bonferroni-corrected pairwise comparisons. The analyses revealed that in the control group, there were significant differences between animal words and tool words (*p* = 0.001), between tool words and food words (*p* = 0.002), and between animal words and each effector-specific type of action word (face-related words: *p* < 0.001; hand-related words: *p* < 0.001; foot-related words: *p* < 0.001). In the ASD group, there were only significant differences between animal words and tool words (*p* = 0.005) and between animal words and foot-related action words (*p* = 0.011), but not between animal words and the other effector-specific action words (hand-related or face-related words), or between tool words and food words.

#### SDT2: Abstract Emotional vs. Abstract Neutral Words

The ANOVA revealed a main effect of *Word Category* both in accuracy (*F*_(1,39)_ = 14.38, *p* < 0.001, $\eta_{\text{p}}^{2}$ = 0.26) and latency (*F*_(1,39)_ = 16.69, *p* < 0.001, $\eta_{\text{p}}^{2}$ = 0.30): in both cases, all participants were faster and more accurate for abstract emotional than abstract neutral words. Furthermore, there was a significant main effect of *Group* in the accuracy analysis (*F*_(1,39)_ = 8.25, *p* = 0.007, $\eta_{\text{p}}^{2}$ = 0.17) with significantly fewer correct responses for all words, regardless of word category, in the ASD group (see Table 4). No significant main effect of *Group* was found in the latency analysis (*F*_(1,39)_ = 3.28, *p* = 0.078, $\eta_{\text{p}}^{2}$ = 0.08). Moreover, there was no significant *Group* × *Word Category* interaction for accuracy (*F*_(1,39)_ = 1.66, *p* = 0.205, $\eta_{\text{p}}^{2}$ = 0.04) or latency (*F*_(1,39)_ = 2.54, *p* = 0.119, $\eta_{\text{p}}^{2}$ = 0.06), suggesting no particular category-specific deficit specific to either group.

### Correlations Between Clinical Data and Semantic Decisions

Pearson correlations were performed between neuropsychological tests, clinical scales, and latency and accuracy data from the semantic decision tasks. The results showed a positive correlation in the ASD group between AQ scores and the overall TAS-26 score (*r* = 0.674, *p* = 0.002). Furthermore, in the ASD group, there was a positive correlation between AQ scores and the MOSES-Test (*r* = 0.766, *p* < 0.001). Regarding the EQ, a negative correlation between AQ and the EQ scores in the autistic group (*r* = −0.499, *p* = 0.03) corroborated previous research, where higher scores on the AQ were associated with lower scores on the EQ. However, there was no significant correlation between any of these tests and the accuracy or latency of semantic judgments for any particular word category.

## Discussion

This study aimed to elucidate the relationship between semantic processing, motor skills and clinical variables in autistic individuals and IQ-matched neurotypical controls. In line with previous findings of action word deficits (Moseley et al., 2013), a significant Group × Word Category interaction was found for accurate data and revealed that autistic participants were significantly less accurate than typically-developing controls when processing words associated with actions. Importantly and in contrast, the ASD group performed as accurately as controls when making semantic decisions about object-related words. This category-specific deficit in action-semantic processing, seen here in another motor-impaired group alongside those noted previously (Boulenger et al., 2009; Bak and Chandran, 2012; Fernandino et al., 2013a,b; Cardona et al., 2014; Kemmerer, 2014; Desai et al., 2015), might be interpreted in terms of an underlying dysfunction of the neuronal action-perception links (Rizzolatti and Fabbri-Destro, 2010; Moseley et al., 2013) suggested to underlie semantic processing (Pulvermüller and Fadiga, 2010; Moseley and Pulvermüller, 2018). Abnormalities in the circuits connecting motor regions to perisylvian language cortices would result in difficulties recognizing or understanding those words which especially draw on these links for the motor programs supporting conceptual knowledge: namely, in the first instance, action words (for a comprehensive review, see Moseley and Pulvermüller, 2018). It is important to note the specificity of this action-semantic processing deficit in the present and the previous study (Moseley et al., 2013), which speaks against the assumption of a more generic semantic language impairment in ASD, which might have been reflected by main effects of Group in SDT1 (see below for discussion of SDT2). Previous studies suggest that the weakness that some clinical groups show in processing action-related stimuli is related to the differing semantic content of action-words and object-related words, rather than their differing grammatical roles (Pulvermüller and Fadiga, 2010; Moseley and Pulvermüller, 2018).

In support of the notion of an underlying action-motor problem in ASD, we found evidence for impaired motor skills in the ASD group compared to controls: in the Purdue Pegboard Test, the ASD group showed reduced hand motor skills when placing pegs in a board with the left hand, the right hand, and with both hands simultaneously. Interestingly, when a complex assembly of different objects with both hands was required, control participants and individuals with ASD performed equally well. Besides fine motor skills, the assembly task tests for bimanual coordination and executive function: our results may suggest that our autistic sample were able to compensate for deficits in unimanual fine motor skills by good performance on bimanual coordination. Although executive dysfunction in autism is assumed to be evident in everyday functioning, it is difficult to capture experimentally in tests with low ecological validity (Kenworthy et al., 2008; Wallace et al., 2016) and poor sensitivity (Demetriou et al., 2018). “Executive function” is a term which encapsulates many higher-level processes, and autistic people tend to show a somewhat inconsistent performance of executive difficulties and executive sparing, which is affected by sample differences in age, gender, IQ (where, notably, our study included only individuals with IQ in the normal range), by common comorbidities such as depression, anxiety and ADHD, and by task features such as complexity, whether tasks are open-ended or more structured (Demetriou et al., 2018) or even whether they measure cognitive performance vs. overt manifestations of difficulties (Albein-Urios et al., 2018)^1^. It is highly likely that the lack of executive impairment seen in our data belies significant difficulties in everyday life (Wallace et al., 2016).In this context, it seems not especially surprising that the autistic sample in our study did not appear impaired on the TMT Parts A and B, where they were compared with normative data from typically-developing participants in the same age range (Tombaugh, 2004). In contrast to previous studies (Hill and Bird, 2006), individuals with ASD in our study performed well on both parts of the TMT, though we were unable to perform a direct comparison to our own control group who did not complete the TMT. Interestingly and specifically relating to the TMT, a stronger performance has been seen in autistic girls and women than autistic boys and men (Bölte et al., 2011; Lehnhardt et al., 2016). This may furthermore explain a lack of group differences in our sample of men *and* women.

To our knowledge, this study is the first one to employ a semantic decision task with abstract emotional and abstract but emotionally neutral words. Based on previous data demonstrating cortical hypoactivation in the motor and limbic cortex in individuals with ASD when processing emotion words (Moseley et al., 2015) and data from patients with motor lesions (Dreyer et al., 2015), we expected to find evidence for impaired processing of abstract emotional words but not for emotionally neutral abstract words; these, like action words, would draw on motor systems for meaning (Moseley et al., 2012) and thus be especially impaired in our participants with movement impairments. Our data did not confirm this prediction but revealed that the ASD group, in general, showed less accurate and slower performance than typically-developing controls, irrespective of these two-word categories. One possible explanation of this finding could be due to the fact that the SDT2 task (abstract emotional words vs. abstract neutral words) was more difficult than the SDT1 task (action vs. object words). This might have led to a lower and more heterogeneous performance in the SDT2 task in both groups, reducing statistical power and thus working against the emergence of a statistically significant Group × Word category interaction.

Correlation analyses calculated between neuropsychological and clinical tests and accuracy and reaction time for semantic decisions did not reveal any statistically significant relationships, including (most notably for this study) a lack of relationship between movement impairments (in both the Purdue Test AND the MOSES-Test) and reaction times and accuracy for those word categories hypothesized to depend most on motor systems: action words and abstract emotional words. As such, our original hypothesis, that autistic deficits in motor skills would be functionally associated with impairments in action-semantic processing, was not statistically supported by the data. This is unexpected given the relationship between motor hypoactivity and impaired action word processing seen previously (Moseley et al., 2013). This previous study in autism, as well as reports from other patient groups with diseases or lesions of the motor system (Boulenger et al., 2009; Bak and Chandran, 2012; Cardona et al., 2014; Kemmerer, 2014), suggest the functional importance of the motor system for optimal action word processing; the studies above also indicate a functional role for motor systems for abstract emotional words (Moseley et al., 2012, 2015; Dreyer et al., 2015) though this proposition has not yet accrued the same degree of empirical support. For action words, at least, simulation studies and studies of novel action word learning have been able to demonstrate the involvement and importance of motor systems in acquiring an action vocabulary. The fact that action and emotion word processing deficits were not related to motor dysfunction appears to speak against this interpretation. However, an interesting possibility is whether the deficits in hand dexterity shown here by the Pegboard Test may have been so specific that they did not correlate with errors to action words which ranged in effector-specificity, as the overall action word category included not only hand-related action words that might correspond with the motor programs employed by the Purdue Pegboard Test, but also those denoting motor programs of the feet and face. The same point could be made regarding emotion words, which foremost tend to be related to actions of the face (Moseley et al., 2012). A more thorough investigation might, as such, include a wider battery of motor tests and a larger sample size with greater power. It is also notable that autistic individuals may, to some extent, be able to compensate for impaired motor systems by recruiting other areas for semantic word processing (Moseley and Pulvermüller, 2018). This may be another reason for the lack of an association, and ultimately, studies would benefit from marrying multiple methodologies: imaging during language testing, *and* motor skills testing.

A notable limitation of our study is the fact that semantic differences between action and object words were confounded by uncontrolled differences in grammatical class: action words were all verbs, while object words were nouns which could have confounded our data. As such, it could be argued that autistic participants had a general deficit across the grammatical category of verbs. Though this study cannot speak to this possibility, our previous investigation in autistic participants found a double dissociation *within* the grammatical category of verbs between words with emotional content and those without (Moseley et al., 2015). Analysis of carefully orthogonalized word categories does indeed suggest that action and object words diverge along the semantic as opposed to grammatical line (Moseley and Pulvermüller, 2014), though dissociations between nouns and verbs as grammatical categories might appear as emergent properties of the more fundamental difference in action and object associations. The primacy of the semantic as opposed to grammatical dissociation has been supported by a number of studies (Barber et al., 2010; Vigliocco et al., 2011; Kemmerer et al., 2012; Fargier and Laganaro, 2015; Lobben and D’Ascenzo, 2015; Popp et al., 2016; Zhao et al., 2017; Vonk et al., 2019), though others reflect both semantic *and* grammatical divisions (Yudes et al., 2016; Yang et al., 2017). We would as such doubt that our findings reflect a general verb deficit in autism, but as debate surrounding the amodal vs. modal organization of language continues, we cannot speak conclusively on this matter.

Another point of note is that one of our subcategories of object words, tool words, is known to elicit activity in motor systems that has been associated with the action affordances of these objects (Chao and Martin, 2000; Carota et al., 2012). Including this more action-related subcategory within our superordinate object-word category might, therefore, have been problematic. In an attempt to exclude the possible contribution of action associations from tool words in our object word category, we ran a secondary analysis excluding tool words, which did not lead to a different pattern of results. As such, the autistic impairment seen for action words was impervious to the presence of tool words in the object word category, but along with tighter control over the grammatical confound of action/verbs and object/nouns, future studies may wish to exclude tool words within superordinate object word categories.

Whilst none of the motor or clinical tests correlated with the semantic language tasks, several other relationships of interest were observed which corresponded with previous research in autism. First, a significant correlation between the severity of autistic symptoms (as measured by the AQ) and the severity of alexithymia (as measured by the TAS-26) was obtained in our autistic participants. This finding suggests that a higher number of autistic traits is associated with greater alexithymia, and is in line with other research that has shown high comorbidity between ASD and alexithymia (Lombardo et al., 2007; Milosavljevic et al., 2016; Kinnaird et al., 2019). Our ASD participants had significantly higher overall scores on all scales of the TAS-26 in comparison to TD controls. Scale 1 of the TAS-26 measures difficulties in identifying feelings, scale 2 measures difficulties in describing (communicating) feelings, and scale 3 measures externally-orientated thinking.

A high degree of consistency was seen between our findings and previous literature on the AQ, the EQ, and the SQ-R: namely, that autistic participants had lower scores on the EQ and that empathy scores decreased as autistic traits increased (as in Baron-Cohen and Wheelwright, 2004; Wheelwright et al., 2006); and that as in previous studies, autistic individuals tend to score highly in systemizing (Baron-Cohen et al., 2003; Wheelwright et al., 2006). This pattern, overall, confirms the empathizing-systemizing account of autism (Baron-Cohen, 2009), and is consistent with that seen in very large samples (Baron-Cohen et al., 2014).

Our self-developed MOSES questionnaire evaluates problems in gross motor skills in daily life (e.g., catching a ball, riding a bicycle, descending stairs, standing on one leg). The ASD group scored significantly higher than controls on this self-report questionnaire, indicating gross motor deficits that corroborate the fine deficits seen in the Purdue Pegboard Test. Furthermore, there was a strong positive correlation between overall AQ scores and the MOSES questionnaire which implies that the degree of autistic traits may correspond to the severity of motor deficits in everyday life situations. Many studies have shown deficits in gross motor skills in individuals with ASD (Leary and Hill, 1996; Jansiewicz et al., 2006; Dziuk et al., 2007; Ming et al., 2007), and many studies have likewise shown a relationship between increased severity of autistic symptomatology and greater motor dysfunction (Papadopoulos et al., 2012; MacDonald et al., 2013, 2014; Travers et al., 2013, 2015; Stevenson et al., 2017; Uljarević et al., 2017; for review, see Moseley and Pulvermüller, 2018). Notably, the MOSES test in our study assessed how participants subjectively *perceived* their own gross motor skills. It is interesting that ASD participants’ perception of their own deficits in gross motor function is consistent with the poorer scores in objective assessments of gross motor skills described in previous studies, and that as in previous studies, a relationship exists between motor deficits and autistic symptomatology, even when the former is self-reported.

Finally, this study possesses limited generalizability within the autism spectrum, due to the fact that only autistic adults without intellectual disability were included. Hence, these findings cannot be generalized to minimally-verbal adults, those with intellectual disability, or to children with ASD. Moreover, although the sample size in the present study is similar compared to other behavioral studies on autism, the results require confirmation in future studies with a larger clinical group.

## Conclusion

Our study corroborates previous findings that autistic individuals show specific difficulties in semantic processing of action words; there was no evidence for differential semantic processing deficits for any other word category. Furthermore, our findings revealed deficits in fine motor skills as well as in self-reported gross motor behavior in autistic adults without intellectual disability. The results might be interpreted on the basis of impaired functional (or structural) connections within the motor cortex that hinders the formation of action-perception circuits which may be crucial for storing semantic concepts. The lack of a significant correlation between motor skills in ASD and deficits for action (and indeed emotion words) did not support the notion of a direct functional-behavioral link between motor performance and semantic processing of these words, but the study leaves open several possible interpretations. Further investigation is thus needed to corroborate the hypothesized functional relationship between motor deficits and impairments in processing words which imply motor regions.

## Data Availability

The datasets generated for this study are available on request to the corresponding author.

## Ethics Statement

This study was carried out in accordance with the recommendations of the Charité Ethics Committee with written informed consent from all subjects. All subjects gave written informed consent in accordance with the Declaration of Helsinki. The protocol was approved by the Charité Ethics Committee.

## Author Contributions

JH contributed to the study design, recruitment and testing of participants, data analysis and writing of the manuscript. BM contributed to the study design, recruitment of participants, data analysis and writing of the manuscript. RM contributed to the study design and writing of the manuscript. SR contributed to the recruitment and testing of participants and writing of the manuscript.

## Conflict of Interest Statement

The authors declare that the research was conducted in the absence of any commercial or financial relationships that could be construed as a potential conflict of interest.

## Footnotes

^1^Indeed, with reference to heterogeneity in task performance, it is important to note that although our autistic sample showed motor deficits in the majority of conditions in the Purdue Pegboard Test, other findings range from an absence of any impairments (Lai et al., 2012), impairments across the board (Barbeau et al., 2015), or inconsistent profiles contradictory to our sample (for instance, poorer performance in the assembly and right-handed condition, but not in the left-handed and simultaneous bimanual condition (Thompson et al., 2017). Again, it should be noted that motor skills are likewise affected by participant characteristics such as autistic symptom severity, IQ, language development and age, and the influence of sex is so far unknown (Moseley and Pulvermüller, 2018).
