# Supplementary material for: Action Semantic Deficits and Impaired Motor Skills in Autistic Adults Without Intellectual Impairment
Source: Front Hum Neurosci. 2019 Jul 25;13:256. doi: 10.3389/fnhum.2019.00256 (PMC6669914; doi:10.3389/fnhum.2019.00256)
Supplement: Supplementary file 1 [file Table_1.DOCX]

Supplementary Material

**1. MOSES Test (Mo**tor **S**kills in **E**veryday **S**ituations)

| 1. | My handwriting is legible. | I completely  agree | I slightly agree | I slightly disagree | I completely disagree |
| --- | --- | --- | --- | --- | --- |

| 2. | I have difficulties in keeping my balance while walking. | I completely  agree | I slightly agree | I slightly disagree | I completely disagree |
| --- | --- | --- | --- | --- | --- |

| 3. | I can easily catch or throw a ball. | I completely  agree | I slightly agree | I slightly disagree | I completely disagree |
| --- | --- | --- | --- | --- | --- |

| 4. | I have difficulties in cutting with scissors. | I completely  agree | I slightly agree | I slightly disagree | I completely disagree |
| --- | --- | --- | --- | --- | --- |

| 5. | I accidentally drop items. | I completely  agree | I slightly agree | I slightly disagree | I completely disagree |
| --- | --- | --- | --- | --- | --- |

| 6. | I have no difficulties riding a bike. | I completely  agree | I slightly agree | I slightly disagree | I completely disagree |
| --- | --- | --- | --- | --- | --- |

| 7. | I often trip. | I completely  agree | I slightly agree | I slightly disagree | I completely disagree |
| --- | --- | --- | --- | --- | --- |

| 8. | I have difficulties in climbing stairs. | I completely  agree | I slightly agree | I slightly disagree | I completely disagree |
| --- | --- | --- | --- | --- | --- |

| 9. | I have difficulties in standing on one leg. | I completely  agree | I slightly agree | I slightly disagree | I completely disagree |
| --- | --- | --- | --- | --- | --- |

| 10. | I often bump against something. | I completely  agree | I slightly agree | I slightly disagree | I completely disagree |
| --- | --- | --- | --- | --- | --- |

| 11. | I like doing sports. | I completely  agree | I slightly agree | I slightly disagree | I completely disagree |
| --- | --- | --- | --- | --- | --- |

| 12. | I had several accidents as a child or as an adult. | I completely  agree | I slightly agree | I slightly disagree | I completely disagree |
| --- | --- | --- | --- | --- | --- |
