# Supplementary material for: Action Semantic Deficits and Impaired Motor Skills in Autistic Adults Without Intellectual Impairment
Source: Front Hum Neurosci. 2019 Jul 25;13:256. doi: 10.3389/fnhum.2019.00256 (PMC6669914; doi:10.3389/fnhum.2019.00256)
Supplement: Supplementary file 2 [file Table_2.DOCX]

Supplementary Material

**Means and standard deviations (in brackets) for psycholinguistic and semantic features of word stimuli for SDT 1 and 2.**

|  | Abstract emotional words | Abstract neutral words | Action words | Object words |
| --- | --- | --- | --- | --- |
| Lemma frequency (per million) | \| 33,19 (35,66) \| \| --- \| \|  \| | 32,69 (56,92) | 23,25 (48,70) | 7,34 (7,36) |
| Length (letters) | 6,87 (2,49) | 7,48 (1,41) | 7,24 (1,49) | 5,52 (1,33) |
| Length (syllables) | \| 2,17 (0,70) \| \| --- \| \|  \| | 2,59 (0,50) | 2,12 (0,33) | 1,86 (0,35) |
| Action relatedness | 3,25 (0,56) | 4,92 (0,68) | 6,59 (0,23) | 3,12 (1,80) |
| Mouth relatedness | 3,00 (0,74) | 1,82 (0,59) | 2,99 (2,53) | 1,34 (0,68) |
| Concreteness | 2,28 (0,50) | 2,63 (0,68) | 6,34 (0,27) | 6,95 (0,03) |
| Emotion relatedness | 6,58 (0,28) | 2,01 (0,80) | 1,39 (0,48) | 1,07 (0,15) |
| Arousal | 5,63 (0,71) | 1,92 (0,68) | 1,69 (0,58) | 1,38 (0,49) |
| Valence | 3,17 (2,32) | 4,19 (0,60) | 3,91 (0,45) | 4,03 (0,22) |
| Foot relatedness | 1,21 (0,24) | 1,27 (0,63) | 3,11 (2,55) | 1,15 (0,12) |
